# Supplementary material for: A Nucleus-Targeting WT1 Antagonistic Peptide Encapsulated in Polymeric Nanomicelles Combats Refractory Chronic Myeloid Leukemia
Source: Pharmaceutics. 2023 Sep 12;15(9):2305. doi: 10.3390/pharmaceutics15092305 (PMC10534672; doi:10.3390/pharmaceutics15092305)
Supplement: Supplementary file 1 [file pharmaceutics-15-02305-s001.zip › pharmaceutics-2553650-supplementary.pdf]

Article

A nucleus-targeting WT1 antagonistic peptide encapsulated by polymeric nanomicelles combat refractory chronic myeloid leukemia

Mengting Chen [1,2,#], Xiaocui Fang [1,2,#], Rong Du [1,2], Jie Meng [1,2], Jingyi Liu [1,2], Mingpeng Liu [1,2,3], Yanlian Yang [1,2,*] and Chen Wang [1,2,*]

Supplementary Information

Table S1. Abbreviations involved in the article.

Abbreviation	Name
Abl	the Abelsonmurine leukemia
AP	accelerated phase
Bcr	the breakpoint cluster region
BP	blast phase
CMC	critical micelle concentration
CML	chronic myeloid leukemia
CP	chronic phase
CPPs	cell-penetrating peptides
DEGs	differentially expressed genes
EPR	enhanced permeation and retention
IVIS	*in vivo* imaging system
KEGG	Kyoto Encyclopedia of Genes and Genomes
PBS	phosphate buffered saline
PEG-PE	Distearoyl-*sn*-glycero-3-phosphoethanolamine-n-[methoxy (polyethylene glycol)-2000
TAT	HIV-1 trans-activator of transcription
WT1	Wilms' tumor 1

Table S2. Amino acid sequences of *de novo*-designed peptides

Peptide name	Amino acid sequence
WIP1	NH_2-DQRPSWGGRRPDRRYGRKKRRQRRR-COOH
WIP2	NH_2-DQRRSWGRRRPDRRYGRKKRRQRRR-COOH
WIP3	NH_2-DQRPSWGGRRPDRRGPKKKRKV-COOH
WIP4	NH_2-RRQSNDSTGYRRRNDYGRKKRRQRRR-COOH
WIP5	NH_2-RRDPYQCRFDRRRDDFSDSRRYGRKKRRQRRR-COOH
WIP21	NH_2-DQKKSWGKKKPDKKYGRKKRRQRRR-COOH
WIP22	NH_2-DQHHSWGHHHPDHHYGRKKRRQRRR-COOH
WIP23	NH_2-DQKKGGGKKKPDKKYGRKKRRQRRR-COOH
WIP2V	NH_2-VVGGDQRRSWGRRRPDRRYGRKKRRQRRR-COOH
WIP2F	NH_2-FFGGDQRRSWGRRRPDRRYGRKKRRQRRR-COOH
WIP2W	NH_2-WWGGDQRRSWGRRRPDRRYGRKKRRQRRR-COOH

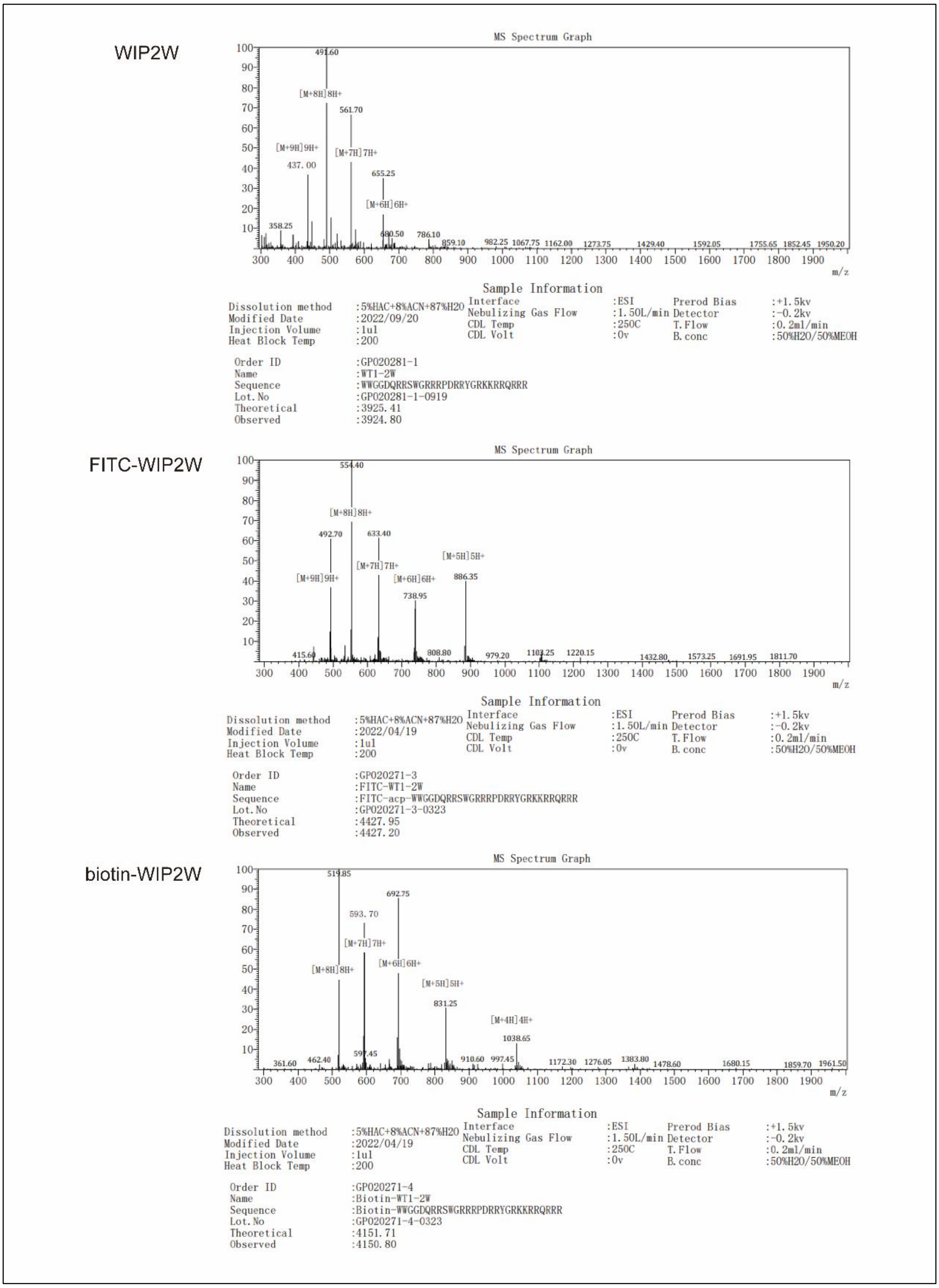

Figure S1. MS Spectrum results of WIP2W, FITC-WIP2W and biotin-WIP2W.

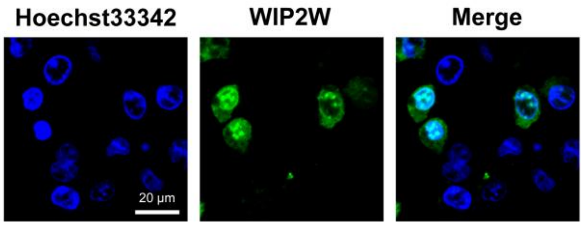

Figure S2. Intracellular localization of WIP2W peptide (10 μM) in K562 cells. Fluorescence images of K562 cells was obtained after incubation with FITC-WIP2W at 37 °C for 3.5 h. The blue fluorescence signifies the nucleus. The green fluorescence represents the FITC-labeled peptide (FITC-WIP2W), and the right figure displays the co-localization (scale bars: 20 μm).

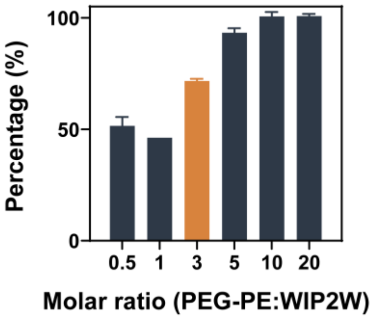

Figure S3. Encapsulation efficiency (%) of WIP2W (10 μM) in PEG-PE micelle at different molar ratios ranging from 0.5: 1 to 20: 1 (PEG-PE: WIP2W) in PBS. Data are presented as mean ± SD (n = 3).

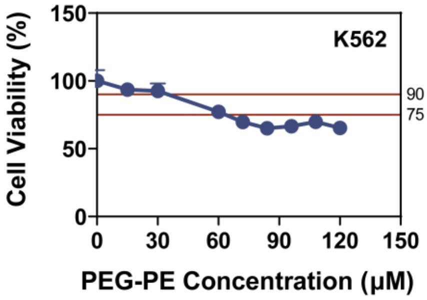

Figure S4. Viability of K562 cells after treatment with PEG-PE (15, 30, 60, 72, 84, 96, 108, and 120 μM) at 37 °C for 24 h was determined using a CCK-8 kit. Data are presented as mean ± SD (n = 6).

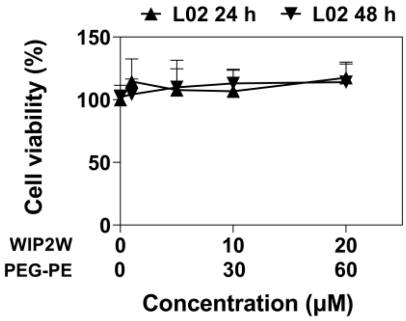

Figure S5. Cytotoxicity of M—WIP2W on L02 cells was evaluated using a CCK-8 kit. L02 cells were treated with M—WIP2W at the indicated concentrations for 24 and 48 h, respectively. Data are presented as mean ± SD (n = 6).

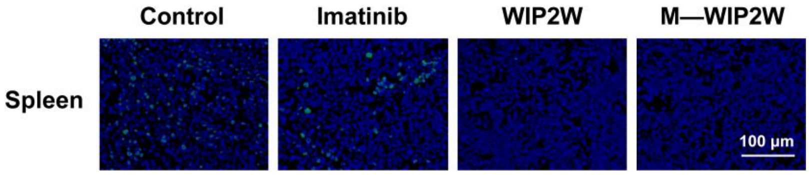

Figure S6. Representative Ki67 staining photomicrographs of the spleen tissues (scale bar: 100 μm).
